# Risk factors for childhood enteric infection in urban Maputo, Mozambique: A cross-sectional study

**DOI:** 10.1371/journal.pntd.0006956

**Published:** 2018-11-12

**Authors:** Jackie Knee, Trent Sumner, Zaida Adriano, David Berendes, Ellen de Bruijn, Wolf-Peter Schmidt, Rassul Nalá, Oliver Cumming, Joe Brown

**Affiliations:** 1 School of Civil and Environmental Engineering, Georgia Institute of Technology, Atlanta, Georgia, United States of America; 2 We Consult, Maputo, Mozambique; 3 Departamento de Geografia, Universidade Eduardo Mondlane, Maputo, Mozambique; 4 Waterborne Disease Prevention Branch, Division of Foodborne, Waterborne, and Environmental Diseases, National Center for Emerging Zoonotic and Infectious Diseases, Centers for Disease Control and Prevention, Atlanta, Georgia, United States of America; 5 Department of Disease Control, London School of Hygiene and Tropical Medicine, London, United Kingdom; 6 Ministério da Saúde, Instituto Nacional de Saúde Maputo, Maputo, Republic of Mozambique; Baylor College of Medicine, UNITED STATES

## Abstract

**Background:**

Enteric infections are common where public health infrastructure is lacking. This study assesses risk factors for a range of enteric infections among children living in low-income, unplanned communities of urban Maputo, Mozambique.

**Methods & findings:**

We conducted a cross-sectional survey in 17 neighborhoods of Maputo to assess the prevalence of reported diarrheal illness and laboratory-confirmed enteric infections in children. We collected stool from children aged 1–48 months, independent of reported symptoms, for molecular detection of 15 common enteric pathogens by multiplex RT-PCR. We also collected survey and observational data related to water, sanitation, and hygiene (WASH) characteristics; other environmental factors; and social, economic, and demographic covariates.

We analyzed stool from 759 children living in 425 household clusters (compounds) representing a range of environmental conditions. We detected ≥1 enteric pathogens in stool from most children (86%, 95% confidence interval (CI): 84–89%) though diarrheal symptoms were only reported for 16% (95% CI: 13–19%) of children with enteric infections and 13% (95% CI: 11–15%) of all children. Prevalence of any enteric infection was positively associated with age and ranged from 71% (95% CI: 64–77%) in children 1–11 months to 96% (95% CI: 93–98%) in children 24–48 months. We found poor sanitary conditions, such as presence of feces or soiled diapers around the compound, to be associated with higher risk of protozoan infections. Certain latrine features, including drop-hole covers and latrine walls, and presence of a water tap on the compound grounds were associated with a lower risk of bacterial and protozoan infections. Any breastfeeding was also associated with reduced risk of infection.

**Conclusions:**

We found a high prevalence of enteric infections, primarily among children without diarrhea, and weak associations between bacterial and protozoan infections and environmental risk factors including WASH. Findings suggest that environmental health interventions to limit infections would need to be transformative given the high prevalence of enteric pathogen shedding and poor sanitary conditions observed.

**Trial registration:**

ClinicalTrials.gov NCT02362932

## Author summary

Enteric pathogens such as bacteria, protozoa, and viruses can cause diarrhea and other longer-term health problems. Poor sanitary conditions, including inadequate sanitation facilities, can lead to contamination of the living environment and higher risk of exposure to and transmission of enteric pathogens. Young children, who are vulnerable to both the short- and long-term health effects of enteric infections, interact with their environment in different ways than older children or adults. In order to limit enteric pathogen transmission among this vulnerable group, we must understand the infection burden and the environmental or sanitation-related factors that are associated with infection. Among a group of children younger than four years old living in low-income neighborhoods of Maputo, Mozambique, we found over 85% tested had ≥1 enteric infection. Children living in environments visibly contaminated with feces were more likely to have an infection than children whose living environments were not visibly contaminated. In contrast, children living in compounds with certain latrine features, including walls and pit covers (potential indicators of build quality), had reduced infection risk. Understanding that these risk factors may play important roles in exposure and transmission in this setting is key to planning effective interventions.

## Introduction

Diarrheal illness is estimated to cause approximately 1.7 million deaths annually and result in over 74 million disability-adjusted life years lost [1], primarily among children in low-and middle-income countries where fecal contamination of the living environment is common. Diarrheal diseases are mostly caused by enteric pathogens, including bacteria, viruses, and protozoa, shed in human and animal feces. These pathogens can be shed in high numbers by both symptomatic and asymptomatic individuals [2]. Although the immediate and longer term health and productivity effects for asymptomatic individuals are unclear [3], persistent asymptomatic infections are associated with environmental enteric dysfunction [4–6] and other conditions, including undernutrition, poor linear growth [7–13], reduced immunogenicity of oral vaccines [14, 15], and cognitive deficits [16–18].

Enteric pathogens are transmitted via several fecal-oral pathways historically defined by the F-diagram [19]. Consumption of contaminated food and water and interaction with fecally contaminated environments have been implicated as dominant transmission pathways for bacterial and protozoan enteric pathogens [20, 21]. While enteric viruses can be transmitted via similar routes, it is posited that person-to-person transmission is also important [22]. Improvements in WASH conditions can reduce risk of diarrheal disease by interrupting transmission pathways. A recent meta-analysis observed reductions in diarrheal disease risk by an average of 67%, 25%, and 30% for water, sanitation, and hygiene interventions, respectively [23]. Sanitation interventions may be more likely to interrupt transmission of protozoa, bacteria, and helminths which are primarily spread via indirect, environmentally mediated pathways than viruses which are often spread via person-to-person transmission [24].

Densely populated, urban, unplanned communities with inadequate sanitary infrastructure represent high-risk settings for exposure to enteric pathogens, though the great majority of sanitation-related exposure and health outcome research has been focused on rural communities where sanitation coverage is lowest and open defecation is common. In the context of the Maputo Sanitation (MapSan) trial [25] (ClinicalTrials.gov Identifier: NCT02362932), we conducted a baseline, cross-sectional survey of compounds (defined as multi-household clusters with shared outdoor space) served by shared latrines. The aim of our study was to estimate prevalence of selected enteric pathogens in stool samples of enrolled children from this cohort, and to identify WASH and other risk factors for enteric infections.

## Methods

### Ethics statement

The head of the compound provided verbal assent for study activities before enrollment of any children within the compound. As children were ≤4 years old at the time of visitation, field enumerators obtained written informed consent from each child’s parent or guardian before enrollment. The study protocol was approved by the Comité Nacional de Bioética para a Saúde (CNBS), Ministério da Saúde (333/CNBS/14), the Ethics Committee of the London School of Tropical Medicine and Hygiene (reference # 8345), and the Institutional Review Board of the Georgia Institute of Technology (protocol # H15160). The associated MapSan trial has been registered at ClinicalTrials.gov (NCT02362932).

### Study design & health outcomes

This cross-sectional study measures enteric infections and key socio-demographic and WASH-related risk factors among children in low-income neighborhoods of Maputo. We defined four outcomes, based on analysis of stool for 15 common enteric pathogens, for our risk factor assessment: (1) detection of any enteric infection, (2) detection of any bacterial infections, (3) detection of any protozoan infections, and (4) detection of any viral infections. We also measured caregiver-reported diarrhea with 7-day recall [26] as a secondary outcome. We defined diarrhea as ≥3 loose or liquid stools in a 24-hour period or any stool with blood [27].

### Study setting

The study sites are located in densely populated, low-income, unplanned neighborhoods of Maputo, Mozambique. Poor sanitary conditions, inadequate infrastructure, environmental conditions including seasonal flooding, and increasingly high population density in these areas has led to a high burden of enteric disease and child mortality [28, 29]. In 2015, an estimated 53% of the urban population in Mozambique (∼4.5 million people) lacked access to basic ‘improved’ sanitation facilities, as defined by the UNICEF/WHO Joint Monitoring Program [30]. In Maputo, approximately 89% of households use onsite waste disposal (10% have access to sewerage; an estimated 1% practice open defecation), and only 26% of fecal waste is safely managed [31]. An estimated 8% of urban sanitation in Mozambique is shared, often among the poorest households in informal neighborhoods [32]. All households in the MapSan trial used shared sanitation facilities that were in poor condition at the time of enrollment.

### Enrollment

Field teams enrolled children and collected baseline data concurrently between February 2015 and February 2016. We enrolled all children who met the following eligibility criteria: (1) the child’s parent or guardian provided written informed consent, (2) the child was 1–48 months of age at the time of enrollment, and (3) the child resided in compounds meeting certain inclusion criteria. Compounds were eligible for enrollment if they were located within a predefined geographic area, were in close proximity to a legal piped water supply, had a minimum number of households (2), and residents shared sanitation in poor condition and had stated demand for improved sanitation. The larger MapSan trial involved additional criteria to select compounds for intervention and details are presented in the supplementary information (S1 Supporting information). Our enrollment period overlapped with the September 2015 rollout of the rotavirus A vaccination program in Mozambique. Children six weeks or younger at the time of rollout and children born after rollout began were eligible for immunization; some children enrolled in our study after September 2015 may have received the vaccination.

### Data collection

Following enrollment, field teams collected data on socio-demographics and WASH-related risk factors using questionnaires and direct observation. Enumerators administered three levels of surveys in each compound with an enrolled child: compound-level, household-level, and child-level. For compound-level surveys, the head of compound or the head of compound’s spouse was the target respondent. For household- and child-level surveys, the child’s mother was the target respondent, though another parent or guardian was eligible to complete the questionnaire. All questionnaires were communicated in either Portuguese or the local language, Changana, as requested by the respondent.

Surveys included socioeconomic and demographic questions such as child age and sex, household assets, caregiver’s education level, and breastfeeding practices. We calculated household wealth using an asset-based wealth index developed for Mozambique [33]. At each level, surveys included direct observations and questions about risk factors of enteric infection, including characteristics of household and compound level water and sanitation, sanitary condition of living spaces, presence of animals within the compound grounds, environmental conditions including flooding patterns, and measures of population density and crowding. We created a composite ‘latrine improvement score’ ranging from 0–4 with one point awarded for the presence of each of the following latrine features: permanent superstructure, tile or masonry slab, drop-hole cover, and ventilation pipe. Similarly, we created a “compound sanitary score” ranging from 0–3 with higher scores indicating poorer sanitary conditions. One point was awarded for each of the following potential risk factors: (1) compound floods during rainy season, (2) leaking or standing wastewater observed by latrine, and (3) feces or soiled diapers observed around compound grounds. Compound-specific population density was defined as the number of people who live in a compound divided by the area of that compound. We measured the area of the compound using high resolution, orthorectified and geolocated satellite imagery. Enumerators equipped with GPS enabled tablets would work with compound residents to identify landmarks and define the shape of a compound on the satellite imagery. We calculated compound area from the shapes and divided the number of compound residents by the calculated compound area to obtain our measure of compound-specific population density. We used rainfall data from the National Oceanic and Atmospheric Administration’s National Centers for Environmental Information (https://www.ncdc.noaa.gov/cdo-web/datatools/findstation) to calculate cumulative rainfall during the 30 days before data collection.

### Sample collection and laboratory analysis

We provided stool collection supplies, including diapers, plastic potties (for older children no longer wearing diapers), and pre-labeled sterile sample bags to the caregiver of each enrolled child. Samples were collected, irrespective of reported symptoms, the following day. If a specimen was not immediately available, caregivers alerted the field team by phone when available. Following collection, samples were stored on cold packs, and transported to the medical parasitology laboratory at the Mozambican Ministry of Health (MISAU/INS) within six hours of collection for storage at -80°C. If a child produced a liquid stool, lab technicians stored a piece of the soaked diaper material (“diaper samples”) at -80°C upon receipt. Stool samples were shipped on dry ice with temperature probes to the Georgia Institute of Technology where they were stored at -80°C until analysis.

We used the Luminex MagPix xTAG Gastrointestinal Pathogen Panel (GPP, Luminex Corp, Austin, TX) to analyze stool samples for the presence of 15 enteric pathogens: *Campylobacter*; *Clostridium difficile*, Toxin A/B; Enterotoxigenic *Escherichia coli* (ETEC) LT/ST; Shiga-like toxin producing *E*. *coli* (STEC) stx1/stx2; *E*. *coli* O157, a serotype of STEC; *Salmonella*; *Shigella*; *Vibrio cholerae*; *Yersinia enterocolitica*; adenovirus 40/41; norovirus GI/GII; rotavirus A; *Giardia*; *Cryptosporidium*; and *Entamoeba histolytica*. The GPP is a stool-based multiplex RT-PCR assay that has been extensively tested for direct detection of enteric infections in a range of countries [34–43]. Per GPP protocol, we pretreated bulk stool samples with 1 mL of ASL stool lysis buffer (Qiagen, Hilden, Germany) and performed nucleic acid extraction for DNA and RNA using the QIAcube HT platform and the QIAamp 96 Virus QIAcube HT Kit (Qiagen, Hilden, Germany). We eluted diaper samples in 2.5 mL of ASL stool lysis buffer. A sterile 10-mL syringe was used to facilitate elution via agitation by taking in and expelling the buffer 5 times. We used 1 mL of the final eluate in the pretreatment step and then proceeded with extraction as previously described. Extracts were stored at 4°C and analyzed by GPP within 24 hours of extraction.

### Data analysis

Sample size for the present study is based on enrollment in the larger MapSan trial. Sample size calculations for the larger MapSan trial have been described previously [25]. To minimize potential bias, we specified the statistical model and variables of interest before beginning the analyses. Details for individual variables used in these analyses—including definitions, coding schemes and proportions of missing values—are available in the supporting information (S1 Table).

We calculated unadjusted and adjusted risk ratios (RRs) and 95% confidence intervals for outcome variables and potential risk factors using generalized estimating equations (GEEs) to fit Poisson regression models with robust standard errors [44]. We used GEEs to account for clustering at the compound level. Outcome variables, including any infection and infection with bacterial, protozoan, or viral pathogens, were defined to identify differences in exposure risks from pathogen groups with different dominant routes of transmission (e.g. person to person versus environment to person). All multivariable models were adjusted for a set of five variables determined *a priori* as contextually important covariates. These variables included child age and sex, breastfeeding practices, caregiver’s education level, and an index of household wealth. We also calculated RRs and aRRs for enteric infections using child age (stratified by age group: 1–11, 12–23, and 24–48 months), sex, breastfeeding practices, and caregiver’s education as the predictors of interest. We ran separate multivariable models for each combination of risk factor and outcome and assessed multicollinearity of multivariable models using the variance inflation factor. We assessed crude and adjusted associations between specific enteric pathogens and diarrheal symptoms as described for the main risk factor analysis.

Our primary analysis focused on complete observations. The proportion of incomplete observations per variable are denoted in supporting information (S1 Table). In parallel with the complete case analysis, we ran all univariable and multivariable models on completed data following multiple imputation (MI) of missing values [45–49]. Details of the MI process are presented in supporting information (S2 Supporting information). Briefly, we performed MI using chained equations (also known as fully conditional specification) to handle missing data [47, 50]. MI models were congenial with previously discussed analysis models and included a fixed effect to account for clustering at the compound level. Auxiliary variables were included in the MI model if they were *a priori* defined as related to either an outcome or predictor, if they were correlated with observed values of an outcome or predictor (r≥0.2), or if they were correlated with missingness of any outcome or predictor variable (r≥0.2) [46]. All statistical analyses were performed with Stata version 14.1 (StataCorp, College Station, TX).

## Results

### Enrollment

Field workers enrolled 519 of the 601 compounds approached regarding participation in the MapSan study. Eighty-two (15.8%) compounds were ineligible for enrollment because they did not have a child <48 months old at the time of visitation. From those 519 compounds, workers enrolled 993 children in 815 households. Field teams administered child-level surveys for 980 of the 993 (99%) enrolled children and collected stool samples from 759 (76%) (S1 Fig: flow diagram of enrollment and data collection activities).

### Sociodemographic characteristics and prevalence of risk factors among study children

The average age of enrolled children was 23 months (Table 1). Approximately 27% (258/944) were <12 months old, while an equal percentage (28%, 266/945) were 12–23 months old, and the remainder (45%, 421/944) were 24–48 months old. Breastfeeding was very common among children 1–11 months old (87%, 224/258), though 31% (82/266) of children 12–24 months were also breastfed. A little over half of child caregivers had completed primary school (527/980). About 17% (163/975) of households met an *a priori* definition of crowding (>3 people per room of living space).

**Table 1:** Baseline measures of demographic, socioeconomic, environmental, and WASH-related exposure variables presented as # participants (%).

	Total n	# (%)
Latrine wall present	974	305 (31)
Drop-hole cover	974	557 (57)
Ventpipe	975	138 (14)
Pedestal or slab	971	361 (37)
Latrine improve index (range 0–4), unitless, mean (SD)	953	1.41 (1.24)
Households per latrine drop-hole	950	
< = 2		171 (18)
3–5		576 (61)
>5		203 (21)
Child feces disposal in latrine	980	289 (29)
Standing water observed	974	71 (7.3)
Waste water observed	974	606 (62)
Feces observed	974	455 (47)
Compound has tendency to flood	974	601 (62)
Compound sanitary score index, unitless, mean (SD)	974	1.71 (1.06)
Drinking water tap on compound grounds	976	757 (78)
Any animal present	993	645 (65)
Dog present	993	76 (7.7)
Ducks or chickens present	993	131 (13)
Cat present	993	550 (55)
Household floor is covered	975	917 (94)
>3 Persons per room (household crowding)	975	163 (17)
Child Age (days), mean (SD)	967	662 (390)
Child sex, female	967	500 (52)
Any breastfed	980	316 (32)
Caregiver completed primary education	980	527 (54)
Wealth index (unitless), mean (SD)	976	43.7 (10.2)

Almost all study children lived in a household that had access to a latrine in the compound (98%, 956/973) and most had access to latrines (61%, 576/950) shared by 3–5 households (median = 4). About half of children had latrines with drop-hole covers (57%, 557/974), 37% (361/971) had a masonry or ceramic slab or pedestal, while only 31% (305/974) had a formal superstructure (made of bricks or cement blocks), and 14% (138/975) had a vent pipe. Sanitary conditions of compounds were poor: 62% (606/974) of study children lived in compounds with wastewater leaking from in or around a latrine and 47% (455/974) lived in compounds where feces or soiled diapers were visible around the grounds. Disposal of child feces into a latrine was common for children 24–48 months old (57%, 238/421). Feces of children between the ages of 1–23 months, most of whom wore diapers, was less frequently disposed of in a latrine (6.4%, 34/528). Most children lived in study compounds with animals (65%, 645/993), with cats (55%, 550/993) most commonly observed. All study households used piped water as their primary drinking water source and 78% (757/976) of children lived in households with access to a drinking water tap on the compound grounds.

### Prevalence of enteric infections in study children

One or more pathogens were identified in stool samples from 655 (~86%) of the 759 children from whom a sample was collected; most (59%, 445/759) had coinfections (Table 2). Stool samples from 66 (8.7%) children yielded four or more enteric pathogens. The prevalence of coinfection (≥2 infections) increased with age from 33% (69/208) in the youngest age group to 73% (214/293) in the oldest. Most children (76%, 579/759) had a bacterial infection, about half (53%, 402/759) had a protozoan infection, and only 14% (107/759) of children had a viral infection. *Giardia*, *Shigella*, ETEC, *Salmonella*, and norovirus were the most frequently detected pathogens among all children, though prevalence varied with age. Prevalence of any infection, and of bacterial and protozoan infections by themselves, increased with age and were largely driven by the most common bacterial and protozoan infections: *Shigella* and *Giardia*. Prevalence of *Shigella* infection increased from 9% (19/208) in children 1–11 months old to 65% (189/293) of children aged 24–48 months. *Giardia* showed a similar pattern with prevalence increasing from 14% (29/208) among 1–11 month-olds to 75% (219/293) prevalence in 24–48 month-olds. Prevalence of viral infections, largely driven by norovirus GI/GII, was highest among the youngest children (17%, 36/208) and lowest among the oldest children (11%, 33/293). Prevalence of rotavirus was low among all age groups (1–2%). Prevalence of enteric infections was similar among boys and girls with the exception of viral infections which tended to be more frequent in girls (17%, 64/370) than boys (11%, 41/370). Only 13% (126/980) of children were reported to have had diarrhea in the previous week. Reported diarrhea was higher among boys (16%, 74/464) than girls (10%, 50/498) and peaked in children aged 12–23 months (20%, 52/266). Norovirus was the only infection associated with higher risk of reported diarrhea (adjusted RR (aRR): 1.76, 95% CI: 1.03–3.02 adjusted for child age and sex, caregiver education, breastfeeding practices, and household wealth (S2 Table), aRR 1.75, 95% CI: 1.00–3.1 when also adjusted for presence of all other measured pathogens).

**Table 2:** Prevalence and 95% confidence intervals of enteric infections in children <4 years of age measured at baseline.

	All, n = 759	Female, n = 370	Male, n = 367	1–11 months, n = 208	12–23 months, n = 225	24–48 months, n = 293
Any Infection (≥1 infections)	0.86 (0.84–0.89)	0.88 (0.84–0.91)	0.85 (0.81–0.89)	0.71 (0.64–0.77)	0.87 (0.82–0.91)	0.96 (0.93–0.98)
Any Viral Infection	0.14 (0.12–0.17)	0.17 (0.14–0.22)	0.11 (0.08–0.15)	0.17 (0.12–0.23)	0.15 (0.11–0.20)	0.11 (0.08–0.15)
Any Bacterial Infection	0.76 (0.73–0.79)	0.78 (0.74–0.82)	0.74 (0.69–0.78)	0.65 (0.58–0.72)	0.74 (0.68–0.80)	0.84 (0.79–0.88)
Any Protozoan Infection	0.53 (0.49–0.57)	0.52 (0.47–0.57)	0.54 (0.49–0.60)	0.18 (0.13–0.24)	0.53 (0.47–0.60)	0.76 (0.71–0.81)
Number of coinfections						
≥2 infections	0.59 (0.55–0.62)	0.62 (0.56–0.67)	0.55 (0.50–0.60)	0.33 (0.27–0.40)	0.60 (0.53–0.66)	0.73 (0.68–0.78)
≥3 infections	0.27 (0.24–0.31)	0.29 (0.24–0.34)	0.25 (0.21–0.30)	0.13 (0.09–0.19)	0.33 (0.27–0.39)	0.32 (0.27–0.38)
≥4 infections	0.09 (0.07–0.11)	0.10 (0.07–0.14)	0.08 (0.05–0.11)	0.04 (0.02–0.08)	0.14 (0.10–0.19)	0.08 (0.05–0.12)
Bacteria						
*Shigella*	0.44 (0.40–0.48)	0.44 (0.39–0.49)	0.43 (0.38–0.48)	0.09 (0.06–0.14)	0.44 (0.38–0.51)	0.65 (0.59–0.70)
ETEC LT/ST	0.30 (0.27–0.34)	0.32 (0.27–0.37)	0.28 (0.24–0.33)	0.23 (0.18–0.29)	0.37 (0.31–0.44)	0.30 (0.25–0.35)
*Salmonella*	0.21 (0.18–0.24)	0.22 (0.18–0.26)	0.19 (0.15–0.24)	0.29 (0.23–0.36)	0.20 (0.16–0.26)	0.16 (0.12–0.20)
*Campylobacter*	0.08 (0.06–0.10)	0.08 (0.06–0.12)	0.08 (0.05–0.11)	0.10 (0.06–0.15)	0.09 (0.06–0.13)	0.05 (0.03–0.09)
*Clostridium difficile*, Toxin A/B	0.05 (0.03–0.06)	0.05 (0.03–0.08)	0.05 (0.03–0.07)	0.11 (0.07–0.16)	0.04 (0.02–0.08)	0.01 (0.00–0.02)
*Escherichia coli *O157	0.04 (0.03–0.06)	0.05 (0.03–0.08)	0.03 (0.01–0.05)	0.03 (0.01–0.06)	0.04 (0.02–0.08)	0.05 (0.03–0.08)
STEC stx1/stx2	0.02 (0.01–0.03)	0.02 (0.01–0.05)	0.01 (0.00–0.03)	0.01 (0.00–0.04)	0.03 (0.01–0.06)	0.01 (0.00–0.03)
*Yersinia enterocolitica*	0.00 (0.00–0.01)	0.00 (0.00–0.01)	0.00 (0.00–0.01)	0.00 (0.00–0.02)	0.00 (0.00–0.02)	0.00 (0.00–0.01)
*Vibrio cholerae*	0.00 (0.00–0.00)	0.00 (0.00–0.01)	0.00 (0.00–0.01)	0.00 (0.00–0.02)	0.00 (0.00–0.02)	0.00 (0.00–0.01)
Protozoa						
*Giardia*	0.51 (0.48–0.55)	0.51 (0.46–0.56)	0.52 (0.47–0.58)	0.14 (0.10–0.19)	0.53 (0.46–0.60)	0.75 (0.69–0.80)
*Cryptosporidium*	0.03 (0.02–0.05)	0.03 (0.01–0.05)	0.04 (0.02–0.06)	0.05 (0.02–0.09)	0.04 (0.02–0.08)	0.02 (0.01–0.04)
*Entamoeba histolytica*	0.01 (0.00–0.01)	0.00 (0.00–0.01)	0.01 (0.00–0.02)	0.00 (0.00–0.03)	0.00 (0.00–0.02)	0.01 (0.00–0.03)
Virus						
Norovirus GI/GII	0.10 (0.08–0.13)	0.13 (0.09–0.17)	0.08 (0.06–0.12)	0.13 (0.09–0.18)	0.11 (0.07–0.16)	0.08 (0.05–0.12)
Adenovirus 40/41	0.03 (0.02–0.04)	0.05 (0.03–0.07)	0.01 (0.00–0.03)	0.03 (0.01–0.07)	0.03 (0.01–0.06)	0.03 (0.01–0.05)
Rotavirus A	0.01 (0.01–0.02)	0.01 (0.00–0.03)	0.01 (0.00–0.03)	0.01 (0.00–0.04)	0.02 (0.01–0.05)	0.01 (0.00–0.02)
Self-Reported Diarrhea	0.13 (0.11–0.15) n = 980	0.10 (0.08–0.13) n = 498	0.16 (0.13–0.20) n = 464	0.14 (0.10–0.19) n = 258	0.20 (0.15–0.25) n = 266	0.09 (0.06–0.12) n = 421

### Risk of any enteric infection in unadjusted and adjusted models

Risk factors for enteric infection were assessed using generalized estimating equations in unadjusted models and models adjusted for age and sex of child, socioeconomic status, caregiver’s education, and any breastfeeding. Among complete cases (Table 3), presence of a latrine superstructure was associated with 7% reduced risk of any enteric infection in the unadjusted model (risk ratio (RR): 0.93, 95% CI: 0.86–1.00), though the association was attenuated in adjusted models (RR: 0.95, 95% CI: 0.89–1.02). Presence of visible feces or used diapers in the compound was a risk factor in both unadjusted and adjusted models (aRR: 1.07, 95% CI: 1.01–1.14). Compound-specific population density was also associated with higher risk of ≥1 enteric infection; children living in the most densely populated quintile of compounds had a 10% higher risk (aRR: 1.10, 95% CI: 1.00–1.21) of any enteric infection compared with children in the least densely populated compounds. Among *a priori* covariates adjusted for in models, any breastfeeding was associated with a 13% reduced risk of any infection in adjusted models. Child age was positively associated with enteric infection; children in the oldest age group were 1.21 times more likely to have an enteric infection than children in the youngest age category.

**Table 3:** Crude and adjusted risk ratios and 95% confidence intervals for associations of WASH-related risk factors and four measures of enteric infection: Any enteric infection, any bacterial infection, any protozoan infection, and any viral infection. Multivariable models are adjusted for child age and sex, caregiver education, household wealth, and breastfeeding practices.

	Any Enteric Infection		Any Bacterial Infection		Any Protozoan Infection		Any Viral Infection		n	
	RR	aRR	RR	aRR	RR	aRR	RR	aRR	UV	MV
Latrine superstructure	0.93 (0.86–1.00)*	0.95 (0.89–1.02)	0.95 (0.86–1.04)	0.96 (0.87–1.06)	0.80 (0.67–0.94)*	0.86 (0.74–1.01)	0.85 (0.56–1.29)	0.89 (0.58–1.35)	747	704
Drop-hole cover present	0.95 (0.89–1.00)	0.96 (0.90–1.01)	0.90 (0.82–0.98)*	0.90 (0.83–0.99)*	0.93 (0.81–1.07)	0.96 (0.85–1.09)	1.00 (0.69–1.47)	0.92 (0.62–1.36)	740	712
Ventpipe present	1.00 (0.92–1.08)	1.01 (0.93–1.10)	0.98 (0.86–1.12)	0.98 (0.85–1.12)	0.94 (0.74–1.18)	0.98 (0.80–1.21)	1.06 (0.64–1.78)	1.17 (0.72–1.89)	741	713
Pedestal or slab present	0.96 (0.90–1.03)	0.97 (0.91–1.03)	1.01 (0.92–1.10)	1.00 (0.91–1.09)	0.94 (0.79–1.10)	0.92 (0.80–1.07)	1.03 (0.70–1.50)	0.93 (0.63–1.37)	737	709
Latrine improvement score	0.97 (0.95–1.00)*	0.98 (0.96–1.00)	0.97 (0.94–1.01)	0.97 (0.94–1.01)	0.94 (0.88–1.00)*	0.96 (0.90–1.01)	0.99 (0.86–1.14)	0.96 (0.83–1.12)	726	698
HHs sharing latrine									728	685
HH< = 2	Reference	Reference	Reference	Reference	Reference	Reference	Reference	Reference		
3–5 HH	0.95 (0.89–1.03)	0.96 (0.90–1.03)	0.95 (0.86–1.05)	0.97 (0.88–1.07)	0.96 (0.80–1.16)	0.99 (0.83–1.18)	1.00 (0.61–1.62)	0.98 (0.59–1.62)		
> 5 HH	0.93 (0.85–1.02)	0.97 (0.89–1.05)	0.89 (0.78–1.02)	0.93 (0.81–1.06)	0.97 (0.77–1.21)	1.11 (0.90–1.37)	0.95 (0.51–1.77)	0.97 (0.51–1.84)		
Disposal of child feces in latrine	1.16 (1.10–1.22)*	1.01 (0.96–1.06)	1.17 (1.07–1.26)*	1.03 (0.93–1.13)	1.76 (1.55–1.99)*	1.08 (0.94–1.23)	0.78 (0.50–1.22)	0.93 (0.56–1.55)	746	714
Standing water in compound	0.99 (0.90–1.10)	0.99 (0.89–1.10)	0.97 (0.81–1.16)	0.96 (0.80–1.16)	1.13 (0.94–1.36)	1.08 (0.86–1.34)	0.71 (0.35–1.42)	0.75 (0.38–1.48)	747	704
Wastewater in compound	1.05 (0.99–1.12)	1.05 (0.99–1.12)	1.06 (0.97–1.16)	1.06 (0.97–1.16)	1.09 (0.93–1.27)	1.15 (0.99–1.32)	1.10 (0.75–1.63)	1.12 (0.75–1.68)	747	704
Visible feces or used diapers	1.08 (1.01–1.14)*	1.07 (1.01–1.14)*	1.07 (0.98–1.16)	1.08 (0.99–1.17)	1.12 (0.97–1.29)	1.16 (1.01–1.32)*	0.85 (0.58–1.24)	0.95 (0.65–1.39)	747	704
Compound floods when it rains	0.98 (0.92–1.04)	0.99 (0.93–1.05)	0.96 (0.88–1.05)	0.98 (0.90–1.07)	0.92 (0.80–1.07)	0.92 (0.81–1.06)	1.15 (0.78–1.69)	1.23 (0.82–1.84)	747	704
Compound sanitary score	1.02 (0.99–1.05)	1.02 (1.00–1.06)	1.02 (0.98–1.06)	1.02 (0.98–1.07)	1.03 (0.96–1.10)	1.04 (0.98–1.11)	1.01 (0.86–1.19)	1.06 (0.89–1.25)	747	704
Drinking water tap on compound grounds	0.97 (0.91–1.03)	0.97 (0.91–1.03)	0.97 (0.88–1.06)	0.97 (0.88–1.07)	0.88 (0.74–1.03)	0.85 (0.73–0.98)*	0.77 (0.51–1.17)	0.89 (0.58–1.36)	742	714
Any animal in compound	1.02 (0.95–1.08)	1.02 (0.95–1.08)	1.03 (0.94–1.12)	1.04 (0.95–1.13)	0.98 (0.84–1.13)	0.95 (0.82–1.08)	1.41 (0.92–2.18)	1.46 (0.93–2.31)	759	714
Dogs in compound	0.98 (0.89–1.08)	0.98 (0.89–1.08)	1.09 (0.98–1.22)	1.10 (0.98–1.22)	0.83 (0.60–1.15)	0.82 (0.61–1.10)	1.25 (0.65–2.43)	1.24 (0.68–2.25)	759	714
Chickens or ducks in compound	1.02 (0.94–1.10)	0.99 (0.92–1.08)	1.00 (0.89–1.13)	1.00 (0.89–1.13)	1.05 (0.87–1.28)	0.97 (0.83–1.15)	0.93 (0.53–1.64)	0.94 (0.54–1.64)	759	714
Cats in compound	1.03 (0.97–1.09)	1.03 (0.97–1.09)	1.03 (0.95–1.12)	1.04 (0.95–1.13)	1.00 (0.87–1.15)	0.95 (0.86–1.08)	1.33 (0.89–1.98)	1.35 (0.90–2.03)	759	714
HH floor is covered	0.94 (0.84–1.04)	0.97 (0.88–1.08)	0.96 (0.82–1.13)	1.02 (0.86–1.21)	0.84 (0.64–1.10)	0.85 (0.70–1.03)	0.58 (0.34–1.00)*	0.59 (0.31–1.09)	741	713
Household crowding, > 3 persons/room	1.00 (0.93–1.08)	0.98 (0.91–1.06)	1.04 (0.94–1.16)	1.02 (0.91–1.14)	0.88 (0.72–1.07)	0.82 (0.68–0.99)*	1.48 (0.98–2.26)	1.52 (0.95–2.43)	741	713
Compound specific population density									740	695
1 (least dense)	Reference	Reference	Reference	Reference	Reference	Reference	Reference	Reference		
2	1.08 (0.97–1.120)	1.07 (0.96–1.18)	1.05 (0.82–1.21)	1.05 (0.91–1.21)	1.07 (0.85–1.34)	1.09 (0.89–1.34)	1.42 (0.78–2.60)	1.39 (0.73–2.64)		
3	1.06 (0.95–1.17)	1.04 (0.94–1.16)	1.13 (0.99–1.28)	1.13 (0.98–1.29)	1.00 (0.79–1.27)	1.01 (0.81–1.26)	0.04 (0.54–2.02)	1.11 (0.57–2.16)		
4	1.07 (0.96–1.19)	1.05 (0.94–1.17)	1.04 (0.90–1.20)	1.03 (0.88–1.20)	1.07 (0.85–1.35)	1.04 (0.83–1.29)	1.41 (0.73–2.71)	1.35 (0.67–2.73)		
5 (most dense)	1.11 (1.01–1.23)*	1.10 (1.00–1.21)*	1.06 (0.93–1.22)	1.06 (0.92–1.23)	1.01 (0.80–1.28)	1.13 (0.93–1.39)	1.56 (0.83–2.91)	1.44 (0.74–2.78)		
Cumulative rainfall last 30 days, terciles									759	714
1 (least rain)	Reference	Reference	Reference	Reference	Reference	Reference	Reference	Reference		
2	0.98 (0.92–1.05)	0.98 (0.92–1.05)	0.93 (0.84–1.02)	0.92 (0.83–1.02)	1.00 (0.84–1.19)	0.95 (0.81–1.10)	1.06 (0.68–1.66)	1.09 (0.69–1.72)		
3 (most rain)	0.95 (0.88–1.02)	0.94 (0.88–1.02)	0.95 (0.86–1.06)	0.94 (0.84–1.04)	1.04 (0.88–1.23)	0.99 (0.85–1.16)	1.22 (0.77–1.95)	1.35 (0.85–2.14)		
Child age									726	698
1–11 months	Reference	Reference	Reference	Reference	Reference	Reference	Reference	Reference		
12–23 months	1.21 (1.10–1.34)*	1.12 (1.00–1.26)	1.12 (0.99–1.27)	1.02 (0.87–1.19)	2.89 (2.08–4.03)*	2.41 (1.64–3.57)*	0.83 (0.53–1.30)	0.62 (0.35–1.10)		
24–48 months	1.34 (1.22–1.47)*	1.21 (1.07–1.36)*	1.28 (1.14–1.44)*	1.14 (0.97–1.34)	4.20 (3.07–5.75)*	3.20 (2.14–4.80)*	0.63 (0.41–0.98)*	0.48 (0.25–0.93)*		
Child sex, female	1.04 (0.98–1.10)	1.04 (0.99–1.10)	1.06 (0.98–1.16)	1.07 (0.99–1.16)	0.95 (0.83–1.09)	0.98 (0.86–1.11)	1.53 (1.09–2.14)*	1.65 (1.17–2.31)*	737	714
Any breastfeeding	0.78 (0.72–0.85)*	0.87 (0.79–0.96)*	0.81 (0.73–0.89)*	0.93 (0.82–1.05)	0.34 (0.27–0.43)*	0.49 (0.36–0.66)*	1.11 (0.76–1.61)	0.81 (0.47–1.37)	742	714
Caregiver completed primary school	0.95 (0.90–1.01)	0.97 (0.92–1.03)	1.00 (0.92–1.09)	1.03 (0.95–1.13)	0.83 (0.72–0.95)*	0.89 (0.79–1.01)	1.17 (0.83–1.65)	1.18 (0.82–1.70)	746	714

*p<0.05

Risk factors for the any infection were also assessed by multiple imputation (S3 Table) and results were consistent with complete case analysis (Table 3).

### Risk of bacterial infection in unadjusted and adjusted models

Risk factors for any bacterial infection were assessed as previously described. Among complete cases (Table 3), presence of a drop-hole cover in the latrine was associated with reduced risk of any bacterial infection (aRR: 0.90, 95% CI: 0.83–0.99). Among *a priori* covariates, any breastfeeding was associated with 19% reduced risk of bacterial infection in the unadjusted model but was not associated with bacterial infection risk in the adjusted model. Despite increasing prevalence of any bacterial infection with age, we found no association between age and bacterial infection in adjusted models. Results from multiple imputation models were consistent with models limited to complete cases (S3 Table).

### Risk of protozoan infection in unadjusted and adjusted models

Among complete cases (Table 3), presence of a latrine superstructure was associated with 20% reduced risk of any protozoan infection in the unadjusted model but was only marginally associated with reduced risk in the adjusted model (aRR: 0.86, 95% CI: 0.74–1.01). In adjusted models, presence of visible feces or used diapers was associated with higher risk of protozoan infection (aRR: 1.16, 95% CI: 1.01–1.32). Household crowding, as well as presence of a drinking water tap on the compound grounds, were associated with reduced risk of protozoan infection in adjusted models only (aRR: 0.85, 95% CI: 0.73–0.98 and aRR: 0.82, 0.68–0.99).

Among *a priori* covariates included in all models, any breastfeeding was associated with reduced risk of protozoan infection in both unadjusted and adjusted models (aRR: 0.49, 95% CI: 0.36–0.66). Caregiver completion of primary school was associated with 17% reduced risk of protozoan infection in the unadjusted model but was only marginally associated in the adjusted model (aRR: 0.89, 95% CI: 0.79–1.01). Age was a risk factor for protozoan infection; children in the 12–23 month and 24–48 month age groups had a 2.41 (1.64–3.57) and 3.20 (2.14–4.80) times higher risk of protozoan infection, respectively, than children aged 0–11 months (Table 3).

Among multiple imputation models, most results were in agreement with those in models limited to only complete cases (S3 Table). The presence of visible feces or used diapers around the compound grounds was not associated with increased risk of protozoan infection in unadjusted or adjusted multiple imputation models.

### Risk of viral infection in unadjusted and adjusted models

Viral infections were not associated with any of the risk factors assessed in adjusted complete case analysis (Table 3). Household crowding (presence of >3 persons per room) was only marginally associated with risk of any viral infection in adjusted models (aRR: 1.55, 95% CI: 0.95–2.43). Among *a priori* covariates, sex was a predictor of viral infection, with girls at higher risk of infection than boys (aRR: 1.65, 95% CI: 1.17–2.31). Children in the oldest age group (24–48 months) had 52% reduced risk of any viral infection compared with the youngest age group (1–11 months).

Results from multiple imputation models were consistent with results from models limited to only complete cases (S3 Table). Among risk factors in multiple imputation models, household crowding was a risk factor in the unadjusted model (RR: 1.55, 95% CI: 1.04–2.32), but not in the adjusted model. Sex remained a risk factor for viral infection in both unadjusted and adjusted MI models.

## Discussion

We observed a high prevalence of enteric infection, including coinfections, among study children yet most children lacked diarrheal symptoms. The prevalence of enteric infection, but not reported diarrhea, increased with age though pathogen-specific age-related patterns varied. We found some independent WASH or environmental risk factors to be associated with enteric infection, though magnitudes of specific associations were often small. In this setting where burden of disease was high and sanitary conditions were poor, pathogen acquisition, symptomology, and the duration of carriage (colonization), may be driven by multiple interdependent risk and protective factors, including acquired immunity.

These results are consistent with findings from other studies of enteric infection in resource-constrained but predominantly rural settings in Africa and elsewhere. The Global Enteric Multicenter Study (GEMS) site in the rural district of Manhiça, Mozambique identified one or more enteric pathogens in 85% of stools from children with moderate-to-severe diarrhea (MSD) and 76% of stools from control children (without diarrhea in the 7 days preceding enrollment) [51]. Similar trends were observed in the Etiology, Risk Factors, and Interactions of Enteric Infections and Malnutrition and the Consequences for Child Health and Development Project (MAL-ED) study sites where 77% diarrheal and 65% of non-diarrheal stool samples were positive for ≥1 enteric pathogen [52]. Studies using the GPP for enteric pathogen detection in similar settings in Ghana and Côte d’Ivoire have also found high prevalence of enteric infection among both symptomatic and asymptomatic children [34, 35].

Compared with enteric infection, the prevalence of caregiver-reported diarrhea was low. We observed a decrease in caregiver-reported diarrhea in children aged 24–48 months compared with the younger age strata, similar to the pattern observed for viral infections. Decreases in reported diarrhea follows a trend observed in historic data of hospital admissions for acute diarrheal episodes among young children in Mozambique [51]. Though we could not formally calculate attributable fractions for etiologic agents of reported diarrhea with these data, we note that norovirus GI/GII was the only enteric pathogen associated with reported diarrhea. This is consistent with findings from the MAL-ED study sites where norovirus GII had one of the highest attributable fractions of diarrhea in children <2 years old [52]. In contrast with reported diarrhea and viral infection, prevalence of bacterial and protozoan infections tended to increase with age, though patterns varied by pathogen. The high prevalence observed here, especially in older children, could be due to the poor clearance and accumulation of persistent enteric infections over time [53] or could be a result of a high rate of reinfection due to frequent pathogen exposure [54]. As children age and become increasingly mobile they interact with their environment more, potentially leading to high exposures to fecal contamination and increased enteric infection [55].

While the overall prevalence of enteric pathogens was similarly high among our study and sites in GEMS and MAL-ED, there were differences in the frequency of detection of specific enteric pathogens. *Giardia* (51%), *Shigella* (44%), ETEC (30%), *Salmonella* (21%) and norovirus GI/GII (10%) were the most frequently detected pathogens in this cohort of children. *Giardia*, rotavirus, *Cryptosporidium*, *E*. *histolytica*, and enteroaggregative *E*. *coli* (EAEC) were the most common pathogens detected among cases and controls at the GEMS-Manhiça site, just 80 kilometers north of our study sites [51]. Across all MAL-ED sites, the most frequently detected pathogens in diarrheal and non-diarrheal stools were *Campylobacter*, *Giardia*, EAEC, and norovirus GII [52]. Notably, even though our data collection occurred largely before the rollout of the rotavirus vaccine in Mozambique in September 2015, we detected almost no rotavirus in our study population. This is in stark contrast to findings from the GEMS-Manhiça site where rotavirus was deemed one of the principal causative agents of MSD and was detected in up to 18% of controls [51]. To further interrogate this difference, we tested the 8 rotavirus GPP-positive specimens and 84 randomly selected rotavirus GPP-negative specimens for the presence of rotavirus using the Premier Rotaclone (Meridian Bioscience, Cincinnati, OH, USA) in-vitro diagnostic fecal antigen enzyme-linked immunosorbent assay (ELISA) [56]. Using the ELISA results as the reference, we calculated the GPP to have 100% sensitivity and 100% specificity for detection of rotavirus A antigen in our fecal specimens. The variations in detection frequencies of enteric pathogens across these studies could be due to differences in detection methods or may suggest that pathogen profiles vary across even limited geographical distances. Molecular reanalysis of the GEMS specimens yielded higher detection frequencies of many bacterial pathogens than the original culture-based methods [57]. However, the GEMS reanalysis did not substantially change detection of *Cryptosporidium* or rotavirus, highlighting potential geographic differences.

Results from this risk factor analysis are consistent with previous studies identifying the build quality or physical characteristics of latrines as factors for increased risk of infection exposure [58]; we found presence of a superstructure or a drop-hole cover to be associated with decreased infection risk. We did not identify any association between enteric infection prevalence and the presence of a cleanable slab, however, consistent with previous work from Tanzania [59]. Associations between the physical characteristics of a latrine and enteric infections were observed only for risk of bacterial and protozoan infections. Household crowding was also associated with a reduced risk of protozoan infection, further evidence that transmission of enteric bacterial and protozoan pathogens is likely to be largely environmentally mediated [20, 21, 24]. We did not identify any WASH or environmental variables associated with risk of viral enteric infection. This is consistent with our prior assumption that person-to-person transmission is likely the predominant pathway for viral infection in this setting [25] as has been observed elsewhere under similar conditions [20].

Consistent with previous work, any breastfeeding appeared protective for enteric infection risk in our analysis [60–65]. Adjusted estimates of association show that this observation is primarily driven by protection from *Giardia* infection (RR = 0.50, 95% CI 0.37–0.67); a similar correlation was also observed in the MAL-ED study [63]. Any breastfeeding limits enteric pathogen transmission by eliminating exposure via direct consumption of contaminated food or water.

Maputo, like many cities of sub-Sahara Africa, is rapidly urbanizing [66]. Urbanization may result in higher risk of direct (person-to-person) or indirect (environmentally-mediated) transmission of enteric infection, especially in low-income, unplanned neighborhoods where WASH infrastructure is lacking [67, 68]. Recent studies of population density and enteric infection risk have found mixed results, though most were based in rural areas or less dense urban settings [69–71]. In our study, we observed an association between higher compound-level population density and higher risk of enteric infections.

There are important limitations to this study that qualify our results. First, our *a priori* selection of specific pathogen targets and our methods for stool sample analysis present key constraints to interpretation. The GPP tests for 15 of the most common enteric pathogens including bacteria, viruses, and protozoa, but this is a sub-set of all enteric infections and therefore an incomplete accounting of current infections. For example, the GPP does not detect EAEC, a pathogen commonly detected in young children in both MAL-ED and the GEMS-Manhiça site [51, 52] and associated with malnutrition [64]. Metagenomics or other primer-independent approaches may have yielded information on additional targets of public health significance. Although detection of pathogens in stool samples was observed to be closely associated with age–suggesting persistent infections or frequent reinfection–we cannot make conclusions about either duration of infections or shedding or about the potential for rapid clearance and reinfection based on a single stool specimen. Detection of an enteric pathogen in stool can represent symptomatic or asymptomatic infection, pathogen carriage due to colonization of the gut, or simply passage due to recent exposure. Further, certain pathogens may be shed for weeks after clinical symptoms of infection have abated, and the onset or absence of symptoms following infection can depend on factors related to the environment, host, or pathogen strain of interest [53]. The GPP was designed to aid in diagnosis of enteric infections and the relatively high limits of detection (2.2x10^2^–3.75x10^6^ CFU or copies/mL stool) [72] largely exceed the known infectious doses for target pathogens. This suggests that enteric pathogen detections via the GPP may primarily represent active infection (symptomatic or asymptomatic) or long or short term colonization of the intestinal tract. Although detection of enteric pathogens in feces is an unambiguous indication of past exposure and a clear indication that fecal waste from such individuals represents downstream exposure risks, absence of a particular pathogen in stool by the methods we used does not indicate absence of previous exposure to that pathogen. Because the detection limit of the assay we used is relatively high, a negative assay may not necessarily mean that the pathogen is absent in stool. Cross-sectional, end-point RT-PCR analysis of stool samples alone cannot reveal information on time since exposure, etiology of symptomatic infections, intensity of infections, health implications of infections, or infectivity of pathogens shed in stool. Enteric infections are on the causal pathway between exposures and all downstream health impacts of WASH, including diarrheal disease and environmental enteric dysfunction, but they should be considered an intermediate outcome of uncertain clinical significance.

Second, the study population and the study setting, though diverse across some variables, was characterized by a limited range of WASH conditions. All participating households had access to shared sanitation without safe excreta management–a key criterion used in determining eligibility for the MapSan trial–and so exposures were likely to be high across our study sites. This lack of heterogeneity of WASH conditions may have limited our ability to observe variation in risk attributable to specific exposures.

Third, certain inclusion criteria may limit the generalizability of our findings. Because our study only included children living in households sharing sanitation in densely populated urban neighborhoods, our results may not represent risks for children in rural areas or in households using private sanitation.

Fourth, our analysis is constrained by missing data for variables, including the outcome. A secondary analysis used multiple imputation (S2 Supporting information and S3 Table) to handle missing values, and these methods are accompanied by different assumptions and limitations. We note, however, that results from the complete case models and estimates from multiple imputation were largely consistent. Finally, our modeling strategy did not include adjustment for multiple comparisons. While it is possible that some of our findings are spurious and due to type I error [73, 74], all variables in this analysis have strong foundations in the literature or plausibility as risk factors for enteric infection.

Overall, we found high prevalence of enteric infection and comparatively low prevalence of reported diarrhea among children <4 years old living in informal neighborhoods of Maputo, Mozambique. Most infections were observed in reportedly asymptomatic children. Prevalence of bacterial and protozoan infection increased with child age and is likely due to variations in exposure profiles as children become more mobile. Certain sanitation facility characteristics were associated with decreased risks of enteric infection, though the magnitude of these associations was small. The importance of effective sanitation increases where prevalence of enteric infections is high: fecal wastes in such settings present elevated exposure risks, potentially driving burdens of infection and disease higher. Strategies to interrupt this cycle of infection and exposure risk should limit the possibility of exposure to excreta, including through multiple pathways of transmission.

## Disclaimer

The findings and conclusions in this report are those of the authors and do not necessarily represent the official position of the U.S. Centers for Disease Control and Prevention.

## Supporting information

S1 Checklist STROBE checklist. (DOCX)

S1 Supporting information Description of compound selection criteria for larger MapSan trial. (DOCX)

S2 Supporting information Additional methods for multiple imputation. (DOCX)

S1 Fig Flow diagram of enrollment and data collection activities. (TIF)

S1 Table Definitions and coding schemes for analysis variables. (DOCX)

S2 Table Crude and adjusted risk ratios and 95% confidence intervals for associations of caregiver reported diarrhea and enteric infection. Multivariable models are adjusted for child age and sex, caregiver education, household wealth, and breastfeeding practices.
(DOCX)

S3 Table Crude and adjusted risk ratios from the multiple imputation risk factor analyses for four measures of enteric infection: Any enteric infection, any bacterial infection, any protozoan infection, and any viral infection. Multivariable models are adjusted for child age and sex, caregiver education, household wealth, and breastfeeding practices.
(DOCX)
