# Supplementary material for: Risk factors for childhood enteric infection in urban Maputo, Mozambique: A cross-sectional study
Source: PLoS Negl Trop Dis. 2018 Nov 12;12(11):e0006956. doi: 10.1371/journal.pntd.0006956 (PMC6258421; doi:10.1371/journal.pntd.0006956)
Supplement: S1 Checklist — (DOCX) [file pntd.0006956.s001.docx]

S1 Checklist: STROBE Checklist

|  | Item No. | Recommendation | Page  No. | Relevant text from manuscript |
| --- | --- | --- | --- | --- |
| **Title and abstract** | 1 | (*a*) Indicate the study’s design with a commonly used term in the title or the abstract | 2 | We conducted a cross-sectional survey in 17 neighborhoods of Maputo to assess the prevalence of reported diarrheal illness and confirmed enteric infections in children |
|  |  | (*b*) Provide in the abstract an informative and balanced summary of what was done and what was found | 2 | We analyzed stool from 755 children living in 425 compounds representing a range of environmental conditions. The prevalence of reported diarrhea was 13%. Prevalence of enteric infections was high with 86% of children positive for one or more infections though most were asymptomatic (87%). We found a high prevalence of enteric infections, primarily among children without diarrhea, and weak associations between bacterial and protozoan infections and environmental risk factors including WASH. |
| Introduction | | | |  |
| Background/rationale | 2 | Explain the scientific background and rationale for the investigation being reported | 3-4  4 | Diarrheal illness is estimated to cause approximately 1.3 million deaths annually and result in over 71 million disability-adjusted life years lost [1], primarily among children in low-and middle-income countries where fecal contamination of the living environment is common. Diarrheal diseases are mostly caused by enteric pathogens, including bacteria, viruses, and protozoa, shed in human and animal feces. These pathogens can be shed in high numbers by both symptomatic and asymptomatic individuals [2]. Although the immediate and longer term health and productivity effects for asymptomatic individuals are unclear [3], persistent asymptomatic infections are associated with environmental enteric dysfunction [4-6] and other conditions, including undernutrition, poor linear growth [7-13], reduced immunogenicity of oral vaccines [14, 15], and cognitive deficits [16-18].  Densely populated, urban, unplanned communities with inadequate sanitary infrastructure represent high-risk settings for exposure to enteric pathogens, though the great majority of sanitation-related exposure and health outcome research has been focused on rural communities where sanitation coverage is lowest and open defecation is common. In the context of the Maputo Sanitation (MapSan) trial [25] (ClinicalTrials.gov Identifier: NCT02362932), we conducted a baseline, cross-sectional survey of compounds (defined as multi-household clusters with shared outdoor space) served by shared latrines. |
| Objectives | 3 | State specific objectives, including any prespecified hypotheses | 4 | The aim of our study was to estimate prevalence of selected enteric pathogens in stool samples of enrolled children from this cohort, and to identify WASH and other risk factors for enteric infections. |
| Methods | | | |  |
| Study design | 4 | Present key elements of study design early in the paper | 5 | This cross-sectional study measures enteric infections and key socio-demographic and WASH-related risk factors among children in low-income neighborhoods of Maputo, Mozambique. We defined four outcomes, based on analysis of stool for 15 common enteric pathogens, for our risk factor assessment: (1) detection of any enteric infection, (2) detection of any bacterial infections, (3) detection of any protozoan infections, and (4) detection of any viral infections. We also measured caregiver-reported diarrhea with 7-day recall [26] as a secondary outcome. |
| Setting | 5 | Describe the setting, locations, and relevant dates, including periods of recruitment, exposure, follow-up, and data collection | 5  5 | The study sites are located in densely populated, low-income, unplanned neighborhoods of Maputo, the capital of Mozambique. Poor sanitary conditions, inadequate infrastructure, environmental conditions including seasonal flooding, and increasingly high population density in these areas has led to a high burden of enteric disease and child mortality [28, 29].  Field teams enrolled children and collected baseline data concurrently between February 2015 and February 2016. |
| Participants | 6 | (*a*) *Cohort study*—Give the eligibility criteria, and the sources and methods of selection of participants. Describe methods of follow-up  *Case-control study*—Give the eligibility criteria, and the sources and methods of case ascertainment and control selection. Give the rationale for the choice of cases and controls  *Cross-sectional study*—Give the eligibility criteria, and the sources and methods of selection of participants | 5-6 | We enrolled all children who met the following eligibility criteria: (1) the child’s parent or guardian provided written informed consent, (2) the child was 1 - 48 months of age at the time of enrollment, and (3) the child resided in compounds meeting certain inclusion criteria. Compounds were eligible for enrollment if they were located within a predefined geographic area, were in close proximity to a legal piped water supply, had a minimum number of households (2), and residents shared sanitation in poor condition and had stated demand for improved sanitation. The larger MapSan trial involved additional criteria to select compounds for intervention and details are presented in the supplementary information (S2 Supporting Information). |
|  |  | (*b*) *Cohort study*—For matched studies, give matching criteria and number of exposed and unexposed  *Case-control study*—For matched studies, give matching criteria and the number of controls per case | N/A | N/A |
| Variables | 7 | Clearly define all outcomes, exposures, predictors, potential confounders, and effect modifiers. Give diagnostic criteria, if applicable | 5  6-7  8 | We defined four outcomes, based on analysis of stool for 15 common enteric pathogens, for our risk factor assessment: (1) detection of any enteric infections, (2) detection of any bacterial infections, (3) detection of any protozoan infections, and (4) detection of any viral infections. We also measured caregiver-reported diarrhea with 7-day recall [26] as a secondary outcome. We defined diarrhea as ≥3 loose or liquids stools in a 24-hour period or any stool with blood [27].  Surveys included socioeconomic and demographic questions such as child age and sex, household assets, caregiver’s education level, and breastfeeding practices. We calculated household wealth using an asset-based wealth index developed for Mozambique [33]. At each level, surveys included direct observations and questions about risk factors of enteric infection, including characteristics of household and compound level water and sanitation, sanitary condition of living spaces, presence of animals within the compound grounds, environmental conditions including flooding patterns, and measures of population density and crowding. We created a composite ‘latrine improvement score’ ranging from 0-4 with one point awarded for the presence of each of the following latrine features: permanent superstructure, tile or masonry slab, drophole cover, and ventilation pipe. Similarly, we created a “compound sanitary score” ranging from 0-3 with higher scores indicating poorer sanitary conditions. One point was awarded for each of the following potential risk factors: (1) compound floods during rainy season, (2) leaking or standing wastewater observed by latrine, and (3) feces or soiled diapers observed around compound grounds. Compound-specific population density was defined as the number of people who live in a compound divided by the area of that compound. We measured the area of the compound using high resolution, orthorectified and geolocated satellite imagery. Enumerators equipped with GPS enabled tablets would work with compound residents to identify landmarks and define the shape of a compound on the satellite imagery. We calculated compound area from the shapes and divided by the number of compound residents to obtain our measure of compound-specific population density. We used rainfall data from the National Oceanic and Atmospheric Administration’s National Centers for Environmental Information (https://www.ncdc.noaa.gov/cdo-web/datatools/findstation) to calculate cumulative rainfall during the 30 days before data collection.  Details for individual variables used in these analyses - including definitions, coding schemes and proportions of missing values - are available in supplemental table 1 (S1 Table). |
| Data sources/ measurement | 8* | For each variable of interest, give sources of data and details of methods of assessment (measurement). Describe comparability of assessment methods if there is more than one group | 6  8 | Following enrollment, field teams collected data on socio-demographics and WASH-related risk factors using questionnaires and direct observation. Enumerators administered three levels of surveys in each compound with an enrolled child: compound-level, household-level, and child-level. For compound-level surveys, the head of compound or the head of compound’s spouse was the target respondent. For household- and child-level surveys, the child’s mother was the target respondent, though another parent or guardian was eligible to complete the questionnaire. All questionnaires were communicated in either Portuguese or the local language, Changana, as requested by the respondent.  We used the Luminex MagPix xTAG® Gastrointestinal Pathogen Panel (GPP, Luminex Corp, Austin, TX) to analyze stool samples for the presence of 15 enteric pathogens: Campylobacter; Clostridium difficile, Toxin A/B; Enterotoxigenic Escherichia coli (ETEC) LT/ST; Shiga-like toxin producing E. coli (STEC) stx1/stx2; E. coli O157, a serotype of STEC; Salmonella; Shigella; Vibrio cholerae; Yersinia enterocolitica; adenovirus 40/41; norovirus GI/GII; rotavirus A; Giardia; Cryptosporidium; and Entamoeba histolytica. Per GPP protocol, we pretreated bulk stool samples with 1 mL of ASL stool lysis buffer (Qiagen, Hilden, Germany) and performed nucleic acid extraction for DNA and RNA using the QIAcube HT platform and the QIAamp 96 Virus QIAcube HT Kit (Qiagen, Hilden, Germany). We eluted diaper samples in 2.5 mL of ASL stool lysis buffer. A sterile 10-mL syringe was used to facilitate elution via agitation by taking in and expelling the buffer 5 times. We used 1 mL of the final eluate in the pretreatment step and then proceeded as previously described. Extracts were stored at 4°C and analyzed by GPP within 24 hours of extraction. |
| Bias | 9 | Describe any efforts to address potential sources of bias | 8 | To minimize potential bias, we specified the statistical model and variables of interest before beginning the analyses. |
| Study size | 10 | Explain how the study size was arrived at | 8 | Sample size for the present study is based on enrollment in the larger MapSan trail. Sample size calculations for the larger MapSan trial have been described previously [25]. |

Continued on next page

| Quantitative variables | 11 | Explain how quantitative variables were handled in the analyses. If applicable, describe which groupings were chosen and why | 8 | Details for individual variables used in these analyses - including definitions, coding schemes and proportions of missing values - are available in supplemental table 1 (S1 Table). |
| --- | --- | --- | --- | --- |
| Statistical methods | 12 | (*a*) Describe all statistical methods, including those used to control for confounding | 8-9 | We calculated unadjusted and adjusted risk ratios (RRs) and 95% confidence intervals for outcome variables and potential risk factors using generalized estimating equations (GEEs) to fit Poisson regression models with robust standard errors [44]. We used GEEs to account for clustering at the compound level. All multivariable models were adjusted for a set of five variables determined a priori as contextually important covariates. These variables included child age and sex, breastfeeding practices, caregiver’s education level, and an index of household wealth. We also calculated RRs and aRRs for enteric infections and using child age (stratified by age group: 1-11, 12-23, and 24-48 months), sex, breastfeeding practices, and caregiver’s education as the predictors of interest. We ran separate multivariable models for each combination of risk factor and outcome and assessed multicollinearity of multivariable models using the variance inflation factor. |
|  |  | (*b*) Describe any methods used to examine subgroups and interactions | 9  9 | We also calculated RRs and aRRs for enteric infections using child age (stratified by age group: 1-11, 12-23, and 24-48 months), sex, breastfeeding practices, and caregiver’s education as the predictors of interest.  We assessed crude and adjusted associations between specific enteric pathogens and diarrheal symptoms as described for the main risk factor analysis. |
|  |  | (*c*) Explain how missing data were addressed | 9 | In parallel with the complete case analysis, we ran all univariable and multivariable models on completed data following multiple imputation (MI) of missing values [45-49]. Details of the MI process are presented in the supplementary information (S2 Supporting Information). Briefly, we performed MI using chained equations (also known as fully conditional specification) to handle missing data [47, 50]. MI models were congenial with previously discussed analysis models and included a fixed effect to account for clustering at the compound level. Auxiliary variables were included in the MI model if they were a priori defined as related to either an outcome or predictor, if they were correlated with observed values of an outcome or predictor (r≥0.2), or if they were correlated with missingness of any outcome or predictor variable (r≥0.2) [46]. |
|  |  | (*d*) *Cohort study*—If applicable, explain how loss to follow-up was addressed  *Case-control study*—If applicable, explain how matching of cases and controls was addressed  *Cross-sectional study*—If applicable, describe analytical methods taking account of sampling strategy | N/A | N/A |
|  |  | (*e*) Describe any sensitivity analyses | 9 | Our primary analysis focused on complete observations. The proportion of incomplete observations per variable are denoted in Table 1 of the supplementary information (S1 Table). In parallel with the complete case analysis, we ran all univariable and multivariable models on completed data following multiple imputation (MI) of missing values [45-49] |
| Results | | | | |
| Participants | 13* | (a) Report numbers of individuals at each stage of study—eg numbers potentially eligible, examined for eligibility, confirmed eligible, included in the study, completing follow-up, and analysed | 9 | Field workers enrolled 519 of the 601 compounds approached regarding participation in the MapSan study. Eighty-two (15.8%) compounds were ineligible for enrollment because they did not have a child <48 months old at the time of visitation. From those 519 compounds, workers enrolled 993 children in 815 households. Field teams administered child-level surveys for 980 of the 993 (99%) enrolled children and collected stool samples from 759 (76%). |
|  |  | (b) Give reasons for non-participation at each stage | N/A | N/A |
|  |  | (c) Consider use of a flow diagram |  |  |
| Descriptive data | 14* | (a) Give characteristics of study participants (eg demographic, clinical, social) and information on exposures and potential confounders | 10-12 | The average age of enrolled children was 23 months (Table 1). Approximately 27% (258/944) were <12 months old, while an equal percentage (28%, 265/944) were 12-23 months old, and the remainder (45%, 421/944) were 24-48 months old. Exclusive breastfeeding was very common among children 1-11 months old (87%, 224/258), though 31% (31/265) of children 12-24 months were also exclusively breastfed. A little over half of child caregivers had completed primary school (527/980). About 17% (163/975) of households were crowded (had >3 people per room).Almost all study children lived in a household that had access to a latrine in the compound (98%, 956/973) and most had access to latrines (61%, 576/950) shared by 3-5 households (median = 4). About half of children had latrines with drophole covers (57%, 557/974), 37% (361/971) had a masonry or ceramic slab or pedestal, while only 31% (305/974) had a formal superstructure (made of bricks or cement blocks), and 14% (138/975) had a vent pipe. Sanitary conditions of compounds were poor: 62% (606/974) of study children lived in compounds with wastewater leaking from in or around a latrine and 47% (455/974) lived in compounds where feces or soiled diapers were visible around the grounds. Disposal of child feces into a latrine was common for children 24-48 months old (57%, 238/421). Feces of children between the ages of 1 – 23 months, most of whom wore diapers, was less frequently disposed of in a latrine. Most children lived in study compounds with animals (65%, 645/993), with cats (55%, 550/993) most common. All study households used piped water as their primary drinking water source and 78% (757/976) of children lived in households with access to a drinking water tap on the compound grounds. |
|  |  | (b) Indicate number of participants with missing data for each variable of interest | 9 | The proportion of incomplete observations per variable are denoted in Table 1 of the supplementary information (S1 Table). |
|  |  | (c) *Cohort study*—Summarise follow-up time (eg, average and total amount) | N/A | N/A |
| Outcome data | 15* | *Cohort study*—Report numbers of outcome events or summary measures over time | N/A | N/A |
|  |  | *Case-control study—*Report numbers in each exposure category, or summary measures of exposure | N/A | N/A |
|  |  | *Cross-sectional study—*Report numbers of outcome events or summary measures | 13 | Table 2. Prevalence and 95% confidence intervals of enteric infections in children <4 years of age measured at baseline. Alternative table format with absolute numbers presented in supplementary table 4 (S5 Table). |
| Main results | 16 | (*a*) Give unadjusted estimates and, if applicable, confounder-adjusted estimates and their precision (eg, 95% confidence interval). Make clear which confounders were adjusted for and why they were included | 14-15  8 | Table 3. Crude and adjusted risk ratios and 95% confidence intervals for associations of WASH-related risk factors and four measures of enteric infection: any enteric infection, any bacterial infection, any protozoan infection, and any viral infection. Multivariable models are adjusted for child age and sex, caregiver education, household wealth, and breastfeeding practices.  All multivariable models were adjusted for a set of five variables determined *a priori* as contextually important covariates. These variables included child age and sex, breastfeeding practices, caregiver’s education level, and an index of household wealth. |
|  |  | (*b*) Report category boundaries when continuous variables were categorized | 14-15  8  8 | Table 3. Crude and adjusted risk ratios and 95% confidence intervals for associations of WASH-related risk factors and four measures of enteric infection: any enteric infection, any bacterial infection, any protozoan infection, and any viral infection. Multivariable models are adjusted for child age and sex, caregiver education, household wealth, and breastfeeding practices.  Details for individual variables used in these analyses - including definitions, coding schemes and proportions of missing values - are available in supplemental table 1 (S2 Table).  We also calculated RRs and aRRs for enteric infections using child age (stratified by age group: 1-11, 12-23, and 24-48 months), sex, breastfeeding practices, and caregiver’s education as the predictors of interest. |
|  |  | (*c*) If relevant, consider translating estimates of relative risk into absolute risk for a meaningful time period | N/A | N/A |

Continued on next page

| Other analyses | 17 | Report other analyses done—eg analyses of subgroups and interactions, and sensitivity analyses | 13  14-15  16  16  17  17 | Norovirus was the only the infection associated with higher risk of reported diarrhea (adjusted RR (aRR): 1.76, 95% CI: 1.03 – 3.02 adjusted for child age and sex, caregiver education, breastfeeding practices, and household wealth, (S5 Table), aRR 1.75, 95% CI: 1.00-3.1 when also adjusted for presence of all other measured pathogens).  Table 3. Crude and adjusted risk ratios and 95% confidence intervals for associations of WASH-related risk factors and four measures of enteric infection: any enteric infection, any bacterial infection, any protozoan infection, and any viral infection. Multivariable models are adjusted for child age and sex, caregiver education, household wealth, and breastfeeding practices.  Risk factors for the any infection were also assessed by multiple imputation (S6 Table) and results were consistent with complete case analysis (Table 3).  Results from multiple imputation models [for any bacterial infection] were mostly consistent with models limited to complete cases (S6 Table).  Among multiple imputation models, most risk factors were consistent with those in models limited to only complete cases [for any protozoan infection] (S6 Table). Presence of a latrine superstructure was associated with reduced risk in unadjusted models (RR: 0.84, 95% CI: 0.71-0.98). Neither visible feces nor used diapers and household crowding were not associated with protozoan infection in unadjusted or adjusted models.  Results from multiple imputation models were consistent with results from models limited to only complete cases [for any viral infection] (S6 Table). Among risk factors in multiple imputation models, household crowding was a risk factor in the unadjusted model (RR: 1.55, 95% CI: 1.04-2.32), but not in the adjusted model. Sex remained a risk factor for viral infection in both unadjusted and adjusted MI models. |
| --- | --- | --- | --- | --- |
| Discussion | | | | |
| Key results | 18 | Summarise key results with reference to study objectives | 17 | We observed a high prevalence of enteric infection, including coinfections, among study children yet most children lacked diarrheal symptoms. The prevalence of enteric infection, but not reported diarrhea, increased with age though pathogen-specific age-related patterns varied. We found some independent WASH or environmental risk factors to be associated with enteric infection, though magnitudes of specific associations were often small. |
| Limitations | 19 | Discuss limitations of the study, taking into account sources of potential bias or imprecision. Discuss both direction and magnitude of any potential bias | 21-22 | There are important limitations to this study that qualify our results. First, our a priori selection of specific pathogen targets and our methods for stool sample analysis present key constraints to interpretation. The GPP tests for 15 of the most common enteric pathogens including bacteria, viruses, and protozoa, but this is a sub-set of all enteric infections and therefore an incomplete accounting of current infections. For example, the GPP does not detect EAEC, a pathogen commonly detected in young children in both MAL-ED and the GEMS-Manhiça site [51, 52] and associated with malnutrition [63]. Metagenomics or other primer-independent approaches may have yielded information on additional targets of public health significance. Although detection of pathogens in stool samples was observed to be closely associated with age – suggesting persistent infections or frequent reinfection – we cannot make conclusions about either duration of infections or shedding or about the potential for rapid clearance and reinfection based on a single stool specimen. Detection of an enteric pathogen in stool can represent symptomatic or asymptomatic infection, pathogen carriage due to colonization of the gut, or simply passage due to recent exposure. Further, certain pathogens may be shed for weeks after clinical symptoms of infection have abated, and the onset or absence of symptoms following infection can depend on factors related to the environment, host, or pathogen strain of interest [53]. The GPP was designed to aid in diagnosis of enteric infections and the relatively high limits of detection (2.2x102 – 3.75x106 CFU or copies/mL stool) [71] largely exceed the known infectious doses for target pathogens. This suggests that enteric pathogen detections via the GPP may primarily represent active infection (symptomatic or asymptomatic) or long or short term colonization of the intestinal tract. Although detection of enteric pathogens in feces is an unambiguous indication of past exposure and a clear indication that fecal waste from such individuals represents downstream exposure risks, absence of a particular pathogen in stool by the methods we used does not indicate absence of previous exposure to that pathogen. Because the detection limit of the assay we used is relatively high, a negative assay may not necessarily mean that the pathogen is absent in stool. Cross-sectional, end-point RT-PCR analysis of stool samples alone cannot reveal information on time since exposure, etiology of symptomatic infections, intensity of infections, health implications of infections, or infectivity of pathogens shed in stool. Enteric infections are on the causal pathway between exposures and all downstream health impacts of WASH, including diarrheal disease and environmental enteric dysfunction, but they should be considered an intermediate outcome of uncertain clinical significance.  Second, the study population and the study setting, though diverse across some variables, was characterized by a limited range of WASH conditions. All participating households had access to shared sanitation without safe excreta management – a key criterion used in determining eligibility for the MapSan trial – and so exposures were likely to be high across our study sites. This lack of heterogeneity WASH conditions may have limited our ability to observe variation in risk attributable to specific exposures.  Third, certain inclusion criteria may limit the generalizability of our findings. Because our study only included children living in households sharing sanitation in densely populated urban neighborhoods, our results may not represent risks for children in rural areas or in households using private sanitation.  ThirdFourth, our analysis is constrained by missing data for variables, including the outcome. A secondary analysis used multiple imputation (Supplemental Information) to handle missing values, and these methods are accompanied by different assumptions and limitations. We note, however, that results from the complete case models and estimates from multiple imputation were largely consistent. Finally, our modeling strategy did not include adjustment for multiple comparisons. While it is possible that some of our findings are spurious and due to type I error [72, 73], all variables in this analysis have strong foundations in the literature or plausibility as risk factors for enteric infection. |
| Interpretation | 20 | Give a cautious overall interpretation of results considering objectives, limitations, multiplicity of analyses, results from similar studies, and other relevant evidence | 22 | Prevalence of bacterial and protozoan infection increased with child age and is likely due to variations in exposure profiles as children become more mobile. Certain sanitation facility characteristics were associated with decreased risks of enteric infection, though the magnitude of these associations was small. The importance of effective sanitation increases where prevalence of enteric infections is high: fecal wastes in such settings present elevated exposure risks, potentially driving burdens of infection and disease higher. Strategies to interrupt this cycle of infection and exposure risk should limit the possibility of exposure to excreta, including through multiple pathways of transmission. |
| Generalisability | 21 | Discuss the generalisability (external validity) of the study results | 22 | Third, certain inclusion criteria may limit the generalizability of our findings. Because our study only included children living in households sharing sanitation in densely populated urban neighborhoods, our results may not represent risks for children in rural areas or in households using private sanitation. |
| Other information | |  | | |
| Funding | 22 | Give the source of funding and the role of the funders for the present study and, if applicable, for the original study on which the present article is based | N/A included in submission filing. | This study was funded by the United States Agency for International Development under Translating Research into Action (Cooperative Agreement No. GHS-A-00-09-00015-00) and the Bill and Melinda Gates Foundation. |

*Give information separately for cases and controls in case-control studies and, if applicable, for exposed and unexposed groups in cohort and cross-sectional studies.

**Note:** An Explanation and Elaboration article discusses each checklist item and gives methodological background and published examples of transparent reporting. The STROBE checklist is best used in conjunction with this article (freely available on the Web sites of PLoS Medicine at http://www.plosmedicine.org/, Annals of Internal Medicine at http://www.annals.org/, and Epidemiology at http://www.epidem.com/). Information on the STROBE Initiative is available at www.strobe-statement.org.
