# Supplementary material for: Risk factors for childhood enteric infection in urban Maputo, Mozambique: A cross-sectional study
Source: PLoS Negl Trop Dis. 2018 Nov 12;12(11):e0006956. doi: 10.1371/journal.pntd.0006956 (PMC6258421; doi:10.1371/journal.pntd.0006956)
Supplement: S1 Supporting information — (DOCX) [file pntd.0006956.s002.docx]

S1 Supporting Information: Description of compound selection criteria for larger MapSan trial.

Compound eligibility and enrollment was driven by the requirements of the intervention and was assessed with the following criteria: (1) compounds are located within a predefined geographical area; (2) residents share a sanitation facility/facilities in poor condition; (3) compounds have at least 12 residents; (4) compounds must be in proximity to a legal piped water supply; (5) there is stated demand for and interest in co-funding improved sanitation among compound residents; (6) sites have adequate space for construction; and (7) sites are accessible for construction and pit-emptying activities. Water and Sanitation for the Urban Poor (WSUP), the organization implementing the intervention, selected intervention sites based on the above criteria, though criterion (3) was not always met and some intervention sites had as few as four members at the time of enrollment. We selected control compounds based on WSUP criteria (1), (2), (4), and (5) with criterion (5) presented as a hypothetical to assess eligibility. To the extent possible, we matched control compounds on population and time of enrollment. Control compounds were also required to have an eligible child (less than four years old) at the time of enrollment. Criteria 6 and 7 are only relevant to the intervention arm of the study and were not assessed during control selection and enrollment. For this analysis we treated the intervention and control arms as a single group.
