# Supplementary material for: Risk factors for childhood enteric infection in urban Maputo, Mozambique: A cross-sectional study
Source: PLoS Negl Trop Dis. 2018 Nov 12;12(11):e0006956. doi: 10.1371/journal.pntd.0006956 (PMC6258421; doi:10.1371/journal.pntd.0006956)
Supplement: S2 Supporting information — (DOCX) [file pntd.0006956.s003.docx]

S2 Supporting Information: Additional methods for multiple imputation

We used two MI models for this analysis; the first any infection as the dependent variable and the second included the outcome measures of any bacterial infection, any protozoan infection, and any viral infection. All risk factors and covariates used in the analysis model were included in the MI model to ensure consistency and limit introduction of bias during imputation. Other than the difference in dependent variable, all other components of the MI models were similar. We used MI with chained equations (MICE) to create 50 complete datasets (m=50). We used MICE given its flexibility in handling binary and categorical variables. Logit distributions were used for all binary variables, ordered logistic for categorical, and predictive mean matching (knn=10) for any continuous or semi-continuous variables. We used an inclusive model with 6 auxiliary variables in the MI models given their observed or presumed association with values or missingness of any of the model variables.
