# Supplementary figures and images for: Risk factors for childhood enteric infection in urban Maputo, Mozambique: A cross-sectional study
Source: PLoS Negl Trop Dis. 2018 Nov 12;12(11):e0006956. doi: 10.1371/journal.pntd.0006956 (PMC6258421; doi:10.1371/journal.pntd.0006956)

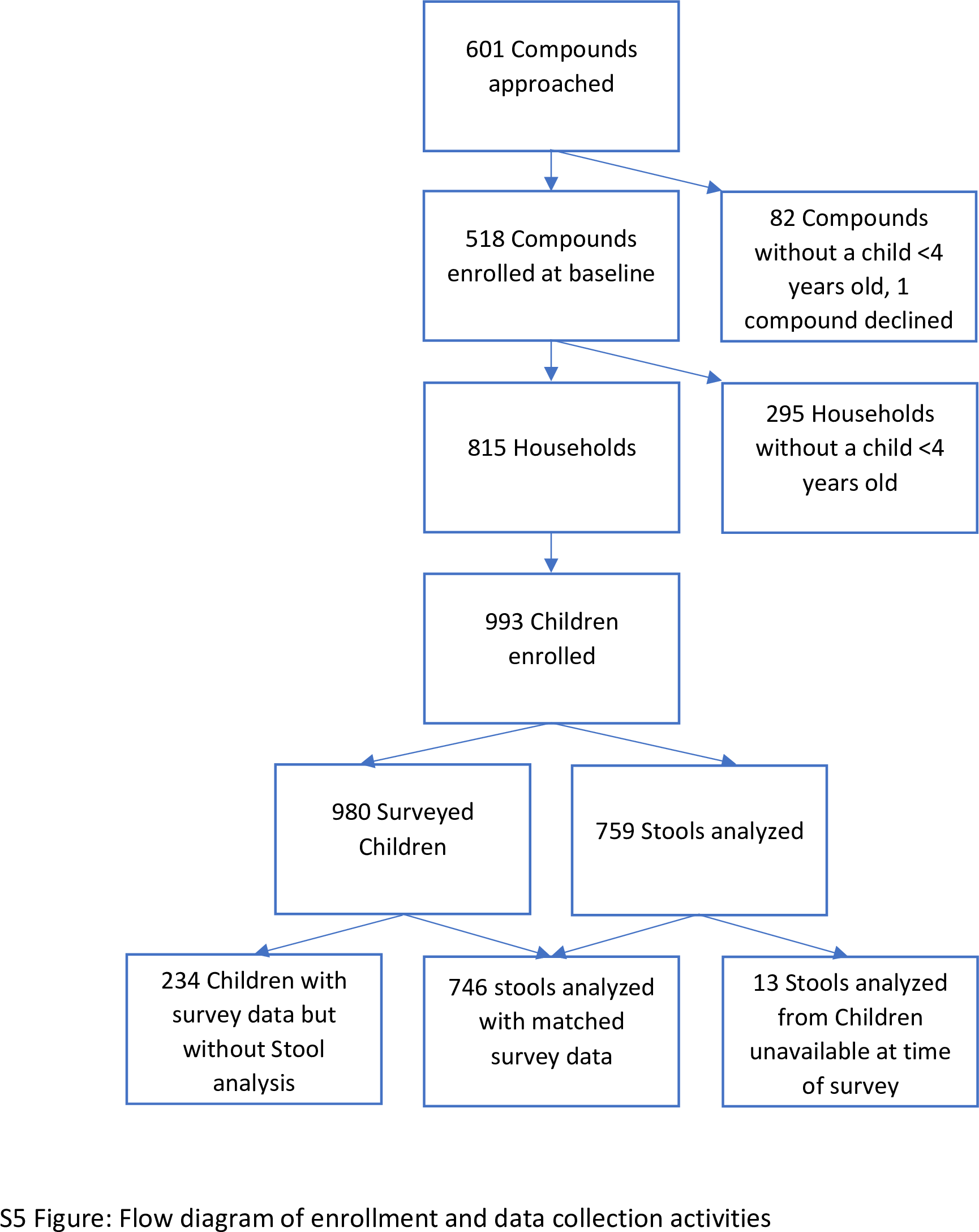

Supplement: S1 Fig — (TIF) [file pntd.0006956.s004.tif]
