# Supplementary material for: Risk factors for childhood enteric infection in urban Maputo, Mozambique: A cross-sectional study
Source: PLoS Negl Trop Dis. 2018 Nov 12;12(11):e0006956. doi: 10.1371/journal.pntd.0006956 (PMC6258421; doi:10.1371/journal.pntd.0006956)
Supplement: S1 Table — (DOCX) [file pntd.0006956.s005.docx]

S1 Table. Definitions and coding schemes for analysis variables.

| **Variable Name** | **% Missing** | **Variable definition and format** |
| --- | --- | --- |
| Latrine superstructure | 1.9 | Binary variable that is 1 when there was a wall around the latrine that provided privacy and security, 0 otherwise |
| Drophole cover present* | 1.9 | Binary variable that is 1 if the latrine drophole was covered, 0 otherwise. |
| Ventpipe present | 1.8 | Binary variable that is 1 if the latrine pit was vented, 0 otherwise. |
| Pedestal or slab present | 2.2 | Binary variable that is 1 if the latrine had a slab or a concrete/masonry pedestal. |
| Composite sanitation score | 4.0 | Ordinal variable ranging from 0-4. One point each awarded for presence of latrine superstructure, drophole cover, ventpipe, or slab/pedestal. |
| HHs sharing latrine | 4.3 | An ordinal variable that was 0 if two or fewer households shared a latrine, 1 if three to five households shared a latrine, and 2 if more than five households shared a latrine. |
| Disposal of child feces in latrine* | 1.3 | Binary variable that is 1 if children's feces were disposed of in a latrine, 0 otherwise (soiled diapers thrown on the trash heap). |
| Standing water in compound | 1.9 | Binary variable that is 1 if the field team observed standing water in the compound at the time of survey, 0 otherwise. |
| Wastewater in compound | 1.9 | Binary variable that is 1 if the field team observed waste water in the compound at the time of survey, 0 otherwise. |
| Visible feces or used diapers | 1.9 | Binary variable that is 1 if the field team observed human feces or soiled diapers in the compound at the time of survey, 0 otherwise. |
| Compound floods when it rains | 1.9 | Binary variable that is 1 if the head of compound reported that the compound had a tendency to flood due to rain, 0 otherwise. |
| Compound hygiene score | 1.9 | Ordinal variable ranging from 0-3. One point each awarded for presence of standing or leaking wastewater in compound, presence of visible feces or soiled diapers on compound grounds, and reported flooding. |
| Drinking water tap on compound grounds | 1.8 | Binary variable that is 1 if there was a water tap within the compound, 0 otherwise. |
| Any animal in compound | 0.0 | Binary variable that is 1 if the head of compound reported that 1 or more animal lived in the compound, 0 otherwise. |
| Dogs in compound | 0.0 | Binary variable that is 1 if the head of compound reported that 1 or more dogs lived in the compound, 0 otherwise. |
| Chickens or ducks in compound | 0.0 | Binary variable that is 1 if the head of compound reported that 1 or more chickens or ducks lived in the compound, 0 otherwise. |
| Cats in compound | 0.0 | Binary variable that is 1 if the head of compound reported that 1 or more cats lived in the compound, 0 otherwise. |
| HH floor is covered | 1.8 | Binary variable that is 1 if the household had some sort of flooring material (such as concrete), 0 otherwise (packed dirt). |
| Household crowding, > 3 persons/room | 1.8 | Binary variable that is 1 if the household had more than three people per room. |
| Compound specific population density | 2.2 | An ordinal variable ranging between 0-4 representing quintiles of compound specific population density (0=least dense, 4=most dense). |
| Cumulative rainfall, terciles | 1. (sampling)   1.7 (survey) | An ordinal variable ranging between 0-2 representing terciles of 30-day cumulative rainfall prior to sampling or survey |
| Age | 2.0 (sampling)  2.6 (survey) | Continuous variable (child age in days at time of sampling and  time of survey) used to create age categories of 1-11 months, 12-23 months, and 24-48 months. |
| Child sex, female | 2.6 | Binary variable that is 1 if child is female, 0 otherwise. |
| Any breastfeeding | 1.3 | Binary variable that is 1 if the child was breastfed at all (exclusively or with complementary foods and liquids), 0 otherwise. |
| Caregiver completed primary school | 1.3 | Binary variable that is 1 if the child's primary caregiver completed primary school, 0 otherwise. |
| Wealth index | 1.7 | Asset based wealth index, continuous variable with theoretical range 0-100. |
