# Supplementary material for: Risk factors for childhood enteric infection in urban Maputo, Mozambique: A cross-sectional study
Source: PLoS Negl Trop Dis. 2018 Nov 12;12(11):e0006956. doi: 10.1371/journal.pntd.0006956 (PMC6258421; doi:10.1371/journal.pntd.0006956)
Supplement: S2 Table — Multivariable models are adjusted for child age and sex, caregiver education, household wealth, and breastfeeding practices. (DOCX) [file pntd.0006956.s006.docx]

S2 Table. Crude and adjusted risk ratios and 95% confidence intervals for associations of caregiver reported diarrhea and enteric infection. Multivariable models are adjusted for child age and sex, caregiver education, household wealth, and breastfeeding practices.

|  | Crude risk ratio, n=652 | Adjusted risk ratio, n=635 |
| --- | --- | --- |
| Any Infection (≥1 infections) | 1.11 (0.65 – 1.89) | 1.32 (0.75 – 2.31) |
| Any Viral Infection | 1.37 (0.82 – 2.29) | 1.34 (0.79 – 2.27) |
| Any Bacterial Infection | 1.19 (0.75 – 1.89) | 1.35 (0.85 – 2.14) |
| Any Protozoan Infection | 0.92 (0.63 – 1.34) | 1.07 (0.70 – 1.61) |
| Bacteria |  |  |
| *Shigella* | 0.95 (0.65 – 1.37) | 1.20 (0.78 – 1.86) |
| ETEC LT/ST | 0.88 (0.58 – 1.34) | 0.88 (0.57 – 1.35) |
| *Salmonella* | 0.96 (0.62 – 1.49) | 0.90 (0.57 - 1.44) |
| *Campylobacter* | 1.07 (0.58 – 1.98) | 0.98 (0.52 – 1.86) |
| *Clostridium difficile*, Toxin A/B | 1.25 (0.59 – 2.65) | 1.11 (0.54 – 2.31) |
| *Escherichia coli*O157 | 1.13 (0.47 – 2.75) | 1.27 (0.51 – 3.15) |
| STEC stx1/stx2 | 0.54 (0.08 – 3.75) | 0.58 (0.08 – 3.97) |
| *Yersinia enterocolitica†* | - | - |
| *Vibrio cholerae†* | - | - |
| Protozoa |  |  |
| *Giardia* | 0.95 (0.65 – 1.38) | 1.11 (0.72 – 1.71) |
| *Cryptosporidium* | 1.56 (0.72 – 3.36) | 1.46 (0.68 – 3.15) |
| *Entamoeba histolytica†* | - | - |
| Virus |  |  |
| Norovirus GI/GII | 1.74 (1.02 – 2.97)* | 1.76 (1.03 – 3.02)* |
| Adenovirus 40/41 | 0.70 (0.20 – 2.41) | 0.40 (0.07 – 2.18) |
| Rotavirus A | 0.79 (0.15 – 4.14) | 0.78 (0.15 – 3.96) |

†Prevalence <0.01. Model not performed. *p<0.05
