# Supplementary material for: Risk factors for childhood enteric infection in urban Maputo, Mozambique: A cross-sectional study
Source: PLoS Negl Trop Dis. 2018 Nov 12;12(11):e0006956. doi: 10.1371/journal.pntd.0006956 (PMC6258421; doi:10.1371/journal.pntd.0006956)
Supplement: S3 Table — Multivariable models are adjusted for child age and sex, caregiver education, household wealth, and breastfeeding practices. (DOCX) [file pntd.0006956.s007.docx]

S3 Table. Crude and adjusted risk ratios from the multiple imputation risk factor analyses for four measures of enteric infection: any enteric infection, any bacterial infection, any protozoan infection, and any viral infection. Multivariable models are adjusted for child age and sex, caregiver education, household wealth, and breastfeeding practices.

|  | Any Infection | | Any Bacterial Infection | | Any Protozoan Infection | | Any Viral Infection | |
| --- | --- | --- | --- | --- | --- | --- | --- | --- |
| n=993 | RR | aRR | RR | aRR | RR | aRR | RR | aRR |
| Latrine superstructure | 0.93 (0.86-1.00)* | 0.94 (0.88-1.01) | 0.94 (0.85-1.04) | 0.95 (0.86-1.04) | 0.82 (0.69-0.97)* | 0.87 (0.75-1.01) | 0.79 (0.52-1.20) | 0.77 (0.51-1.18) |
| Drophole cover present | 0.94 (0.89-1.00) | 0.96 (0.91-1.02) | 0.90 (0.83-0.98)* | 0.92 (0.85-0.99)* | 0.94 (0.83-1.08) | 0.99 (0.88-1.12) | 0.97 (0.68-1.39) | 0.93 (0.65-1.34) |
| Ventpipe present | 0.99 (0.91-1.08) | 1.00 (0.92-1.09) | 0.98 (0.86-1.11) | 0.98 (0.87-1.11) | 0.95 (0.77-1.18) | 0.95 (0.79-1.15) | 1.07 (0.64-1.78) | 1.09 (0.67-1.80) |
| Pedestal or slab present | 0.97 (0.91-1.03) | 0.97 (0.92-1.03) | 1.00 (0.92-1.10) | 1.00 (0.92-1.10) | 0.96 (0.83-1.12) | 0.97 (0.84-1.11) | 0.99 (0.67-1.45) | 0.96 (0.66-1.40) |
| Latrine improvement index | 0.97 (0.95-1.00)* | 0.98 (0.96-1.01) | 0.97 (0.93-1.00) | 0.98 (0.94-1.01) | 0.95 (0.89-1.00) | 0.97 (0.92-1.02) | 0.97 (0.84-1.12) | 0.97 (0.84-1.12) |
| HHs sharing latrine |  |  |  |  |  |  |  |  |
| HH<=2 | Reference | Reference | Reference | Reference | Reference | Reference | Reference | Reference |
| 3-5 HH | 0.95 (0.89-1.03) | 0.97 (0.91-1.04) | 0.95 (0.86-1.06) | 0.98 (0.88-1.08) | 0.96 (0.80-1.16) | 1.02 (0.87-1.20) | 1.00 (0.62-1.61) | 0.99 (0.61-1.61) |
| > 5 HH | 0.94 (0.86-1.03) | 0.98 (0.90-1.06) | 0.90 (0.79-1.03) | 0.95 (0.83-1.07) | 0.98 (0.79-1.22) | 1.11 (0.92-1.35) | 0.94 (0.51-1.72) | 0.93 (0.50-1.72) |
| Disposal of child feces in latrine | 1.15 (1.09-1.22)* | 1.00 (0.95-1.06) | 1.17 (1.08-1.27)* | 1.02 (0.93-1.12) | 1.73 (1.53-1.97)* | 1.07 (0.94-1.22) | 0.79 (0.52-1.20) | 0.95 (0.58-1.57) |
| Standing water in compound | 0.98 (0.87-1.11) | 0.96 (0.86-1.08) | 0.94 (0.77-1.13) | 0.92 (0.77-1.11) | 1.13 (0.93-1.38) | 1.04 (0.87-1.25) | 0.65 (0.29-1.45) | 0.69 (0.31-1.51) |
| Wastewater in compound | 1.06 (0.99-1.13) | 1.05 (0.99-1.12) | 1.07 (0.98-1.16) | 1.07 (0.98-1.16) | 1.10 (0.95-1.28) | 1.09 (0.96-1.25) | 1.05 (0.72-1.53) | 1.05 (0.72-1.52) |
| Visible feces or used diapers | 1.08 (1.02-1.14)* | 1.07 (1.01-1.13)* | 1.07 (0.98-1.16) | 1.06 (0.98-1.15) | 1.12 (0.98-1.28) | 1.08 (0.96-1.22) | 0.84 (0.58-1.23) | 0.88 (0.61-1.28) |
| Compound floods when it rains | 1.00 (0.94-1.06) | 1.00 (0.94-1.06) | 0.98 (0.90-1.07) | 0.98 (0.91-1.07) | 0.95 (0.82-1.10) | 0.94 (0.83-1.07) | 1.19 (0.81-1.74) | 1.20 (0.82-1.76) |
| Compound sanitary score | 1.03 (1.00-1.06) | 1.02 (1.00-1.05) | 1.02 (0.98-1.07) | 1.02 (0.98-1.06) | 1.04 (0.97-1.11) | 1.03 (0.97-1.09) | 1.01 (0.85-1.19) | 1.01 (0.85-1.19) |
| Drinking water tap on compound grounds | 0.98 (0.91-1.04) | 0.97 (0.91-1.03) | 0.97 (0.89-1.06) | 0.97 (0.88-1.06) | 0.90 (0.77-1.06) | 0.86 (0.75-0.99)* | 0.77 (0.51-1.14) | 0.78 (0.53-1.16) |
| Any animal in compound | 1.03 (0.96-1.09) | 1.01 (0.96-1.08) | 1.03 (0.95-1.13) | 1.02 (0.93-1.11) | 0.99 (0.87-1.14) | 0.95 (0.84-1.08) | 1.42 (0.92-2.21) | 1.43 (0.92-2.21) |
| Dogs in compound | 0.96 (0.86-1.08) | 0.96 (0.86-1.07) | 1.08 (0.96-1.22) | 1.07 (0.95-1.21) | 0.83 (0.60-1.15) | 0.80 (0.61-1.06) | 1.27 (0.66-2.42) | 1.19 (0.64-2.22) |
| Chickens or ducks in compound | 1.01 (0.93-1.10) | 0.99 (0.91-1.08) | 1.01 (0.90-1.13) | 0.99 (0.89-1.11) | 1.04 (0.86-1.24) | 0.98 (0.83-1.15) | 0.96 (0.55-1.68) | 0.98 (0.56-1.71) |
| Cats in compound | 1.02 (0.96-1.09) | 1.02 (0.96-1.08) | 1.03 (0.95-1.12) | 1.02 (0.94-1.10) | 0.98 (0.86-1.12) | 0.95 (0.84-1.07) | 1.35 (0.90-2.04) | 1.36 (0.91-2.04) |
| HH floor is covered | 0.94 (0.85-1.04) | 0.98 (0.88-1.08) | 0.97 (0.83-1.14) | 1.03 (0.87-1.21) | 0.87 (0.68-1.12) | 0.88 (0.71-1.09) | 0.54 (0.32-0.91)* | 0.56 (0.30-1.04) |
| Household crowding, > 3 persons/room | 1.00 (0.93-1.08) | 0.97 (0.90-1.05) | 1.04 (0.94-1.15) | 1.01 (0.90-1.12) | 0.86 (0.71-1.04) | 0.83 (0.69-1.00) | 1.56 (1.02-2.40)* | 1.54 (0.96-2.45) |
| Compound specific population density |  |  |  |  |  |  |  |  |
| 1 (least dense) | Reference | Reference | Reference | Reference | Reference | Reference | Reference | Reference |
| 2 | 1.07 (0.96-1.19) | 1.05 (0.95-1.16) | 1.05 (0.91-1.20) | 1.03 (0.90-1.17) | 1.05 (0.86-1.29) | 1.02 (0.84-1.22) | 1.25 (0.70-2.22) | 1.23 (0.69-2.19) |
| 3 | 1.06 (0.96-1.17) | 1.05 (0.95-1.15) | 1.11 (0.97-1.26) | 1.10 (0.97-1.24) | 1.00 (0.80-1.25) | 0.98 (0.81-1.20) | 1.03 (0.56-1.91) | 1.04 (0.56-1.90) |
| 4 | 1.07 (0.97-1.18) | 1.05 (0.95-1.16) | 1.04 (0.90-1.20) | 1.02 (0.89-1.18) | 1.03 (0.83-1.29) | 1.00 (0.82-1.22) | 1.30 (0.69-2.43) | 1.26 (0.67-2.38) |
| 5 (most dense) | 1.10 (1.00-1.21) | 1.09 (1.00-1.20) | 1.06 (0.92-1.22) | 1.05 (0.91-1.21) | 0.98 (0.78-1.23) | 1.02 (0.83-1.25) | 1.43 (0.79-2.57) | 1.32 (0.72-2.41) |
| Cumulative rainfall last 30 days, terciles |  |  |  |  |  |  |  |  |
| 1 (least rain) | Reference | Reference | Reference | Reference | Reference | Reference | Reference | Reference |
| 2 | 0.98 (0.92-1.05) | 0.98 (0.92-1.05) | 0.95 (0.86-1.04) | 0.95 (0.86-1.04) | 1.00 (0.85-1.18) | 0.99 (0.86-1.14) | 1.05 (0.66-1.65) | 1.06 (0.67-1.68) |
| 3 (most rain) | 0.94 (0.88-1.02) | 0.95 (0.88-1.02) | 0.96 (0.86-1.06) | 0.96 (0.87-1.06) | 1.04 (0.88-1.21) | 1.04 (0.90-1.20) | 1.17 (0.74-1.87) | 1.18 (0.74-1.88) |
| Child age |  |  |  |  |  |  |  |  |
| 1-11 months | Reference | Reference | Reference | Reference | Reference | Reference | Reference | Reference |
| 12-23 months | 1.21 (1.10-1.33)* | 1.12 (1.00-1.26)* | 1.13 (1.00-1.28) | 1.05 (0.90-1.21) | 2.74 (1.99-3.78)* | 2.14 (1.49-3.09)* | 0.85 (0.55-1.30) | 0.75 (0.43-1.30) |
| 24-48 months | 1.34 (1.22-1.47)* | 1.21 (1.07-1.37)* | 1.28 (1.14-1.43)* | 1.15 (0.98-1.35) | 4.07 (2.99-5.53)* | 2.89 (1.97-4.24)* | 0.65 (0.42-0.98)* | 0.55 (0.30-1.03) |
| Child gender, female | 1.04 (0.98-1.10) | 1.04 (0.98-1.10) | 1.07 (0.99-1.15) | 1.07 (0.99-1.15) | 0.99 (0.87-1.12) | 0.99 (0.88-1.11) | 1.55 (1.09-2.20)* | 1.53 (1.08-2.17)* |
| Any breastfeeding | 0.79 (0.73-0.85)* | 0.87 (0.79-0.96)* | 0.81 (0.73-0.89)* | 0.92 (0.81-1.04) | 0.34 (0.27-0.43)* | 0.49 (0.38-0.64)* | 1.18 (0.83-1.70) | 0.89 (0.54-1.46) |
| Caregiver completed primary school | 0.95 (0.89-1.00) | 0.98 (0.93-1.04) | 0.99 (0.92-1.08) | 1.03 (0.95-1.12) | 0.84 (0.73-0.96)* | 0.91 (0.79-1.04) | 1.10 (0.77-1.56) | 1.10 (0.78-1.57) |

*p<0.05
